# *Fgf16* Is Required for Specification of GABAergic Neurons and Oligodendrocytes in the Zebrafish Forebrain

**DOI:** 10.1371/journal.pone.0110836

**Published:** 2014-10-30

**Authors:** Ayumi Miyake, Tatsuya Chitose, Eriko Kamei, Atsuko Murakami, Yoshiaki Nakayama, Morichika Konishi, Nobuyuki Itoh

**Affiliations:** Department of Genetic Biochemistry, Kyoto University Graduate School of Pharmaceutical Sciences, Sakyo, Kyoto, Japan; Universitat Pompeu Fabra, Spain

## Abstract

Fibroblast growth factor (Fgf) signaling plays crucial roles in various developmental processes including those in the brain. We examined the role of Fgf16 in the formation of the zebrafish brain. The knockdown of *fgf16* decreased cell proliferation in the forebrain and midbrain. *fgf16* was also essential for development of the ventral telencephalon and diencephalon, whereas *fgf16* was not required for dorsoventral patterning in the midbrain. *fgf16* was additionally required for the specification and differentiation of γ–aminobutyric acid (GABA)ergic interneurons and oligodendrocytes, but not for those of glutamatergic neurons in the forebrain. Cross talk between Fgf and Hedgehog (Hh) signaling was critical for the specification of GABAergic interneurons and oligodendrocytes. The expression of *fgf16* in the forebrain was down-regulated by the inhibition of Hh and Fgf19 signaling, but not by that of Fgf3/Fgf8 signaling. The *fgf16* morphant phenotype was similar to that of the *fgf19* morphant and embryos blocked Hh signaling. The results of the present study indicate that Fgf16 signaling, which is regulated by the downstream pathways of Hh-Fgf19 in the forebrain, is involved in forebrain development.

## Introduction

The forebrain becomes regionally subdivided into the telencephalon and diencephalon during early embryonic brain development in vertebrates. The telencephalon is further subdivided into the rostrally positioned subpallial (ventral) telencephalon and more caudally located pallial (dorsal) telencephalon. The diencephalon is comprised of the hypothalamus, zona limitans intrathalamica (ZLI), ventral thalamus, dorsal thalamus, and pretectum [1]. The regional specification, growth, and differentiation of telencephalic and diencephalic subdivisions are controlled by interactions between secreted signaling molecules. The dorsal region of the telencephalon coordinates growth and patterning via Bone morphogenetic proteins (Bmps) and Wnts [2]. On the other hand, Hedgehog (Hh) signaling is known to be critical for ventral patterning in the forebrain and midbrain [3]–[5]. Fibroblast growth factor (Fgf) signaling has also been implicated in dorsoventral patterning and the regulation of cell proliferation and differentiation in various regions during brain development [1], [6]–[9].

Fgfs comprise a large family of at least 22 members in vertebrates [10]. Of these, *Fgf8* specifies rostral telencephalic fate and represses caudal telencephalic fate in mice and zebrafish [11]–[16]. Furthermore, the ectopic expression of *fgf3* in zebrafish affects the expression of genes that have been implicated in the development of the forebrain [17] and the knockdown of both *fgf3* and *fgf8* functions revealed that *fgf3* and *fgf8* possessed a unique and combinatorial function in regional patterning of the forebrain and hindbrain [7], [18]–[20]. In contrast, an analysis of *Fgf15* knockout mice demonstrated that Fgf15 repressed rostral telencephalic fate [21]. On the other hand, the function of *fgf19*, which is the *Fgf15* orthologue in zebrafish, is known to be essential for development of the ventral region of the telencephalon and diencephalon in zebrafish [8].

*Fgf16*, which was originally identified in the rat heart, is predominantly expressed in the heart at adult stages [22], [23]. *Fgf16* is expressed in the heart, inner ear and brown adipose tissue during embryonic development in mammals [22], [24]–[27]. Three lines of *Fgf16* knockout mice have been reported and their phenotypes may potentially be affected by genetic backgrounds. *Fgf16* knockout mice on a C57BL/6 background exhibited a decrease in the proliferation of embryonic cardiomyocytes and pathophysiological roles in the postnatal heart, whereas the cardiac phenotype of *Fgf16* knockout mice on a 129/B6 background has not yet been examined [25], [27], [28]. These two lines are viable, whereas *Fgf16* knockout mice on a Black Swiss background died at approximately E11.5 [26]. *fgf16* is expressed in zebrafish in the pectoral fin bud and forebrain in addition to the otic vesicle [29]. An analysis of *fgf16* knockdown zebrafish embryos indicated that *fgf16* is an apical ectodermal ridge (AER) factor that is crucial for pectoral fin bud outgrowth [29]. In addition, *fgf16* morphants display morphological abnormalities in the brain. However, these abnormalities have not yet been elucidated in detail.

In the present study, we examined the roles of *fgf16* during brain development in zebrafish. Our results demonstrated that *fgf16* was critical for cell proliferation in the forebrain and midbrain. *fgf16* was also critical for development of the ventral region of the telencephalon and diencephalon, and was implicated in the specification of γ–aminobutyric acid (GABA)ergic interneurons and oligodendrocytes in the telencephalon and diencephalon. On the other hand, *fgf16* was not implicated in the specification of tectal and tegmental fates. *fgf3, fgf8* and *fgf19* have also been shown to be involved in the specification of GABAergic interneurons and oligodendrocytes in the ventral region of the forebrain [8]. Thus, we also examined the crosstalk between *fgf16* and *fgf3*, *fgf8,* and *fgf19* in the forebrain.

## Materials and Methods

### Fish maintenance

Zebrafish (*Danio rerio*) were maintained, according to *The Zebrafish Book* [30]. Embryos were obtained by natural spawning and cultured at 28.5°C in Zebrafish Ringer’s solution. The developmental stages of the embryos were determined by the hours post fertilization (hpf) and morphological features, as described by Kimmel et al. [31]. All animal studies were conducted according to the guidelines of the Institutional Animal Care and Use Committee (IACUC) of Kyoto University Graduate School of Pharmaceutical Sciences (KUGSPS). The animal protocol was approved by the IACUC of KUGSPS; the approved protocol number was 2014-54.

### Whole mount *in* *situ* hybridization

Digoxigenin-labeled RNA probes were synthesized by *in* *vitro* transcription using T7 or SP6 RNA polymerase. The *fgf16* probe was synthesized using the full-length cDNA-containing plasmid. The other probes used were zebrafish *emx1* [32], *tbr1* [33], *dlx2* [34], *shh* [35], *pax6a* [36], *ngn1* [37], *isl1* [38], *otx2* [39], *nkx6.2* [40], *pax7a* [41], *fgf8* [12], *gad1* [42], *slc17a6a* [43], *olig2* [44] and *plp* [44]. Whole mount *in* *situ* hybridization was performed as previously described [45].

### Morpholino and mRNA injection

Morpholino oligonucleotides (MOs) were synthesized by Gene-Tools, LLC (Corvallis, OR). MOs were diluted in Danieau buffer [46]. Universal control MO, *fgf3* MO, *fgf8* MO, *fgf16* MO, and *fgf19* MO have been reported previously [8], [29], [47]. *fgf16* MO1 (5 ng) or universal control MO (5 ng) was injected into the two-cell embryos of zebrafish. *fgf3* MO (10 µg/µl) and *fgf8* MO (20 µg/µl) were injected at a volume of 0.15–0.25 nl into the two-cell embryos. *fgf19* MO was injected at 10 µg/µl into the four central blastomeres of 16-cell embryos.

To construct *fgf16*, full-length *fgf16* cDNA was amplified by PCR and inserted into the vector pCS2+ [48]. Capped *fgf16* mRNA was made by *in* *vitro* transcription using SP6 polymerase (mMESSAGE mMACHINE; Ambion). mRNA was diluted to 0.5 ng/µl with distilled water and injected at a volume of 0.5 nl into 2-cell embryos.

### H3P antibody staining and TUNEL labeling

Proliferating and apoptotic cells were detected using a rabbit polyclonal anti-phosphorylated histone H3 (H3P) (Upstate Biotechnology) antibody and the DeadEndTM colorimetric detection kit (Promega), respectively [8]. For cell counts, the stained embryos were embedded in Technovit 7100 (Heraeus Kulzer, Wehrheim) and cut into 4-µm serial sections. These sections were then counterstained with hematoxylin.

### Immunohistochemistry

Whole mount immunostaining was performed as described previously [49]. The following primary antibodies were used: rabbit anti-GABA (1∶1000; Sigma) [50] and mouse anti-APC (1∶50; Calbiochem) [51]–[53]. We used Alexa Fluor 488 goat anti-rabbit or anti-mouse IgG (1∶200; Molecular Probes) for fluorescent detection.

### Cyclopamine treatments

Cyclopamine (Toronto Chemical) [54] was dissolved at 10 mM in 95% ethanol. Embryos, which were in their chorions, were incubated in cyclopamine diluted to 100 µM in Zebrafish Ringer’s solution starting at the time points indicated. Control embryos were treated simultaneously with an equal volume of 0.95% ethanol (cyclopamine carrier) in Zebrafish Ringer’s solution.

## Results

### Inhibition of *fgf16* functions resulted in defects in brain formation

**Figure 1:**
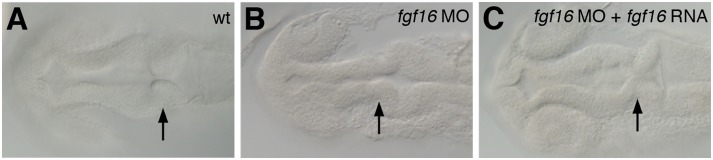
Morphology of the brain in *fgf16* morphants. Dorsal views of wild-type (A), *fgf16* MO-injected (B), and *fgf16* MO- and *fgf16* RNA-injected (C) embryos at 24 hpf. Arrows indicate the MHB constriction.

We previously showed that zebrafish *fgf16* was expressed in the pectoral fin bud and also that the knockdown of *fgf16* function resulted in the absence of fin bud outgrowth at 5 days post-fertilization (dpf) [29]. In addition, the brain structures of *fgf16* morphants exhibited abnormalities at 5 dpf [29]. *fgf16* morphants were morphologically distinguishable from the wild type at 24 hours post-fertilization (hpf). *fgf16* morphants showed morphological abnormalities in the forebrain at 24 hpf (Fig. 1B). Furthermore, *fgf16* morphants were morphologically defective in the formation of midbrain-hindbrain boundary (MHB) constriction and exhibited a failure to evaginate laterally in the midbrain at 24 hpf (Fig. 1B). The gross morphological phenotypes obtained by an injection of either *fgf16* MO1 or *fgf16* MO2 were similar to each other (MO1, n = 78/89 and MO2, n = 79/112). On the other hand, control MO-injected embryos developed normally during embryogenesis [9]. Furthermore, the phenotype was confirmed by RNA rescue experiments. The co-injection of *fgf16* RNA with *fgf16* MO1 rescued the brain defects caused by *fgf16* MO1 (n = 10/13) (Fig. 1C). These results suggested that *fgf16* may be required for normal development in the forebrain and midbrain, and the formation of MHB constriction during neurogenesis.

### Expression pattern of *fgf16* in the brain

**Figure 2:**
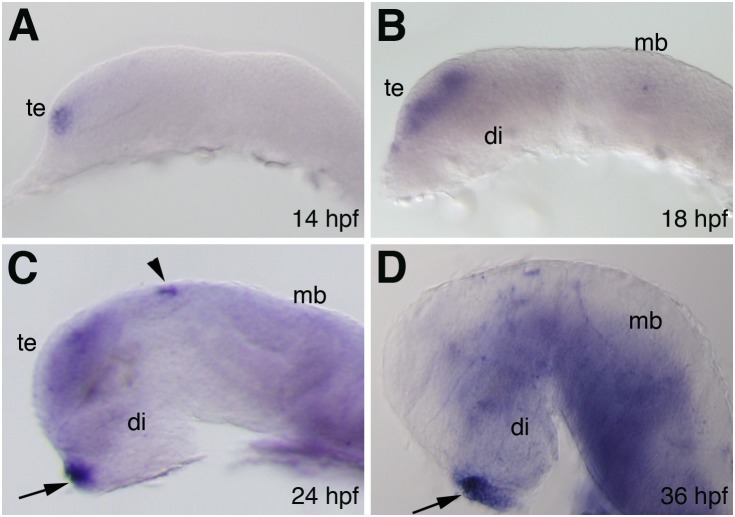
Expression pattern of *fgf16* in the brain during zebrafish embryonic development. (A–D) Expression pattern of *fgf16* in zebrafish embryos at the indicated stages as detected by whole-mount *in* *situ* hybridization. Lateral views with anterior to the left and dorsal to the top. Arrows and arrowheads indicate the pituitary gland and epiphysis, respectively. di, diencephalon; mb, midbrain; te, telencephalon.

*fgf16* is expressed in the brains of zebrafish embryos during 18–36 hpf [29]. However, the expression profile of *fgf16* has not yet been examined in detail in the brain during neural development. We here examined the spatiotemporal expression pattern of *fgf16* in the zebrafish embryonic brain in detail using whole mount *in* *situ* hybridization. The expression of *fgf16* was first detected in the most ventral part of the anterior telencephalon primordium at 14 hpf (Fig. 2A). By 18 hpf, its expression had intensified in the telencephalon and the expression domain had expanded into the dorsal region (Fig. 2B). In addition, *fgf16* was expressed in the diencephalon and midbrain at low levels (Fig. 2B). Its expression was maintained in the forebrain and the midbrain at 24 hpf (Fig. 2C). In addition, the strong expression of *fgf16* was detected in the epiphysis and pituitary gland at 24 hpf (Fig. 2C). The expression of *fgf16* had intensified in the diencephalon and ventral region of the midbrain at 36 hpf and its expression in the telencephalon was markedly decreased (Fig. 2D).

### *fgf16* was required for cell proliferation in the forebrain and midbrain

**Figure 3:**
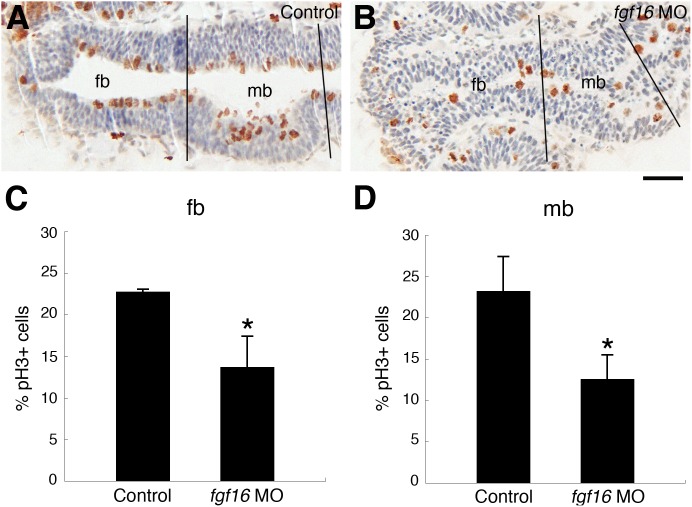
Comparison of cell proliferation and cell death patterns in control embryos and *fgf16* morphants. (A, B) Control embryos (A) and embryos injected with *fgf16* MO (B) were stained using an anti-H3P antibody. Panels show representative horizontal sections of the head region at 24 hpf. (C, D) The percentage of proliferating cells labelled with the anti-pH3 antibody in the forebrain (C) and midbrain (D) of control embryos and embryos injected with *fgf16* MO. Results are the mean ± S.D. for three independent sections from three embryos. The significance of differences in mean values was assessed with the Student’s *t*-test. Asterisks indicate significant differences from the control (*P<0.05). The forebrain (fb) and midbrain (mb) regions, which we defined in the sections, are separated by black lines. Scale bar: 25 µm.

Fgf signaling has been shown to regulate cell proliferation and cell survival in the brains of mice and zebrafish [8], [55], [56]. *fgf16* is also required for cell proliferation in the mesenchyme of fin buds [29]. Therefore, the morphological abnormalities observed in the forebrain and midbrain of *fgf16* morphants at 24 hpf may have been due to a defect in cell proliferation and/or cell survival in these regions. To examine this, we compared the number of mitotic cells in wild-type embryos and *fgf16* morphants. Phosphorylated histone H3 (pH3) was specifically detected in the mitotic cells in mitotic phase (M-phase) [57]. We identified proliferating cells as pH3-positive cells. The rate of pH3-positive cells in the forebrain of *fgf16* morphants was significantly lower than that in wild-type embryos at 24 hpf (Fig. 3A–C). In addition, the rate of pH3-positive cells in the midbrain was significantly decreased in *fgf16* morphants (Fig. 3A, B, D). These results suggested that *fgf16* may promote cell proliferation in the forebrain and midbrain. *fgf16* morphants were also assayed for apoptotic cells via TUNEL labeling at 24 hpf. The number of apoptotic cells in the forebrain and midbrain was slightly higher in the *fgf16* morphants than in the wild-type embryos (n = 6/6)(Fig. S1A, B).

### *fgf16* was required for the development of the subpallial telencephalon and ventral thalamus

**Figure 4:**
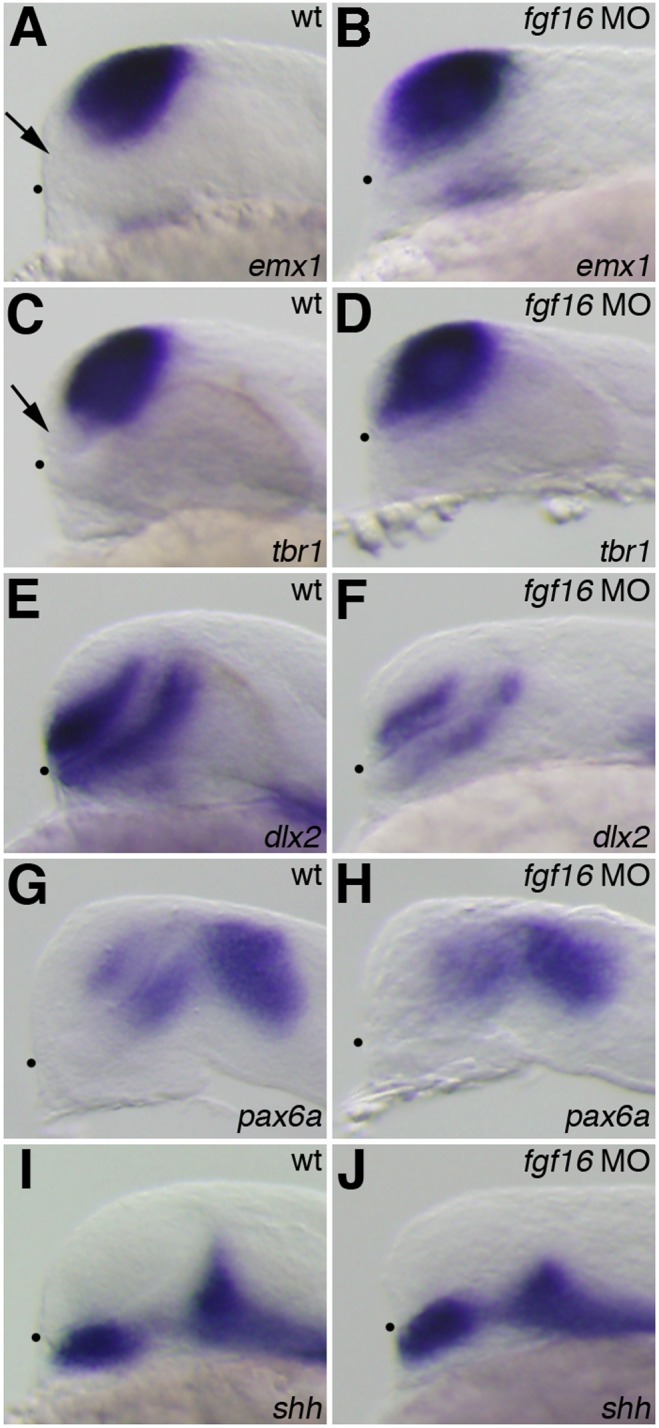
Telencephalic and diencephalic gene expression in the *fgf16* morphants. The expression of *emx1* (A, B), *tbr1* (C, D), *dlx2* (E, F), *pax6a* (G, H), and *shh* (I, J) in wild-type embryos (A, C, E, G, I) and *fgf16* morphants (B, D, F, H, J) at 24 hpf. Arrows in panels A and C indicate the subpallial telencephalon, which was negative for *emx1* or *tbr1*. Dots indicate the boundary between the telencephalon and ventral diencephalon. Lateral views with anterior to the left and dorsal to the top.

**Figure 5:**
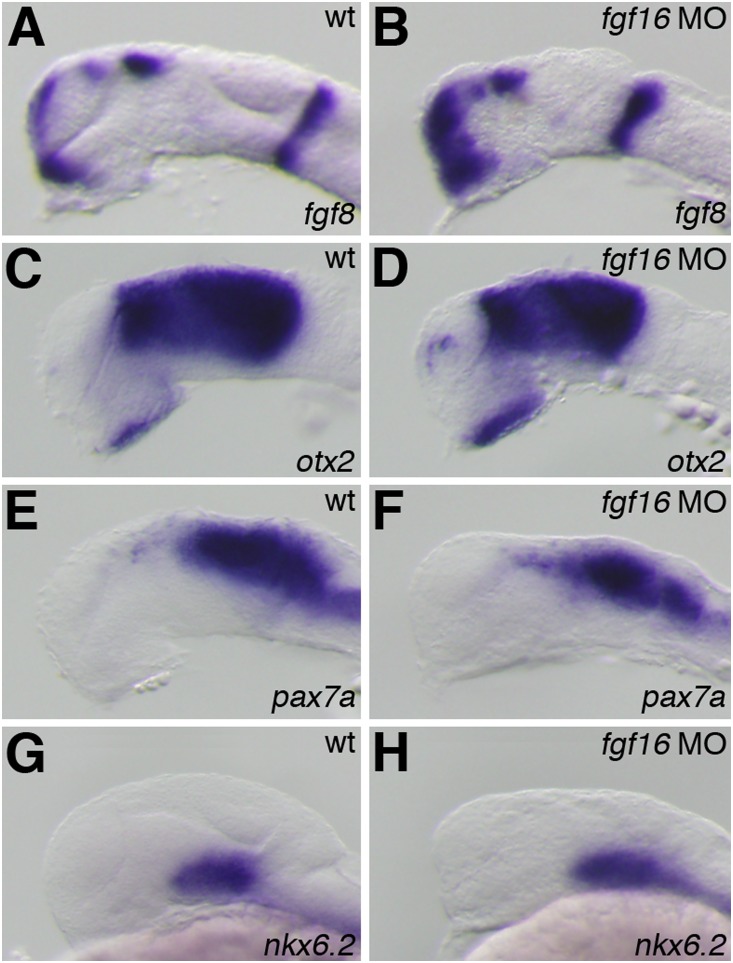
Gene expression in the midbrain and MHB of the *fgf16* morphants. The expression of *fgf8* (A, B), *otx2* (C, D), *pax7a* (E, F), and *nkx6.2* (G, H) in wild-type embryos (A, C, E, G) and *fgf16* morphants (B, D, F, H) at 24 hpf. Lateral views with anterior to the left and dorsal to the top.

*fgf3, fgf8*, and *fgf19* have been implicated in patterning events in the zebrafish forebrain [7], [8], [18]–[20]. Therefore, we investigated whether Fgf16 was also involved in the regionalization of the forebrain. The expression of telencephalon marker genes was analyzed in *fgf16* morphants at 24 hpf. The expression of *emx1*, which is normally detected in the pallial domain of the telencephalon, was observed in the entire region of the telencephalon in *fgf16* morphants (n = 28/32) (Fig. 4A, B). Furthermore, the expression of *tbr1*, which normally occurs in the pallial telencephalon, was also detected in the entire telencephalon in *fgf16* morphants (n = 15/16) (Fig. 4C, D). In contrast to the expression of *emx1* and *tbr1*, that of *dlx2*, which is normally detected in the ventral region of the telencephalon, was reduced in *fgf16* morphants (n = 27/31) (Fig. 4E, F). On the other hand, the expression of *pax6a*, which is normally detected in the telencephalon, was unaffected in *fgf16* morphants (n = 21/21) (Fig. 4G, H). The ectopic expression of *otx2* was detected in the ventral region of the telencephalon in *fgf16* morphants at 24 hpf (n = 13/13) (Fig. 5C, D). In contrast, all control embryos showed normal expression patterns for these genes (data not shown). These results indicated that *fgf16* was required for the development of the subpallial telencephalon.

We also determined whether the inhibition of *fgf16* affected diencephalic specification. In addition to the ventral telencephalon, *dlx2* is normally expressed in the ventral thalamus. The expression of *dlx2* in the ventral thalamus was reduced in *fgf16* morphants at 24 hpf (n = 27/31) (Fig. 4E, F). On the other hand, the expression of *pax6a* and *otx2* in the diencephalon was unaffected in *fgf16* morphants at 24 hpf (n = 21/21 and n = 13/13, respectively) (Fig. 4G, H and 5C, D). We also analyzed the expression of *shh*, which is normally detected in the hypothalamus, ZLI, and floor plate. The ZLI, which is located in the intrathalamic boundary, may locally regulate the development of the ventral and dorsal thalamus through Hh signaling [58], [59]. The expression of *shh* in the ZLI was reduced in *fgf16* morphants (n = 14/14) (Fig. 4I, J). On the other hand, the expression of *shh* in the hypothalamus and floor plate was unaffected in *fgf16* morphants (n = 14/14) (Fig. 4I, J). Thus, these results indicated that *fgf16* was required for the formation of the ZLI and development of the ventral thalamus, but not for the establishment of the dorsal thalamus.

### fgf16 was not required for patterning in the midbrain

The MHB is the most characterized local organizing center and is crucial for induction and patterning in the midbrain [60]–[62]. Fgf8 was previously shown to be required for MHB development and is involved in cell proliferation in the midbrains of chicks [63]. *fgf16* morphants showed morphological abnormalities in the MHB constriction and midbrain. Therefore, to investigate whether *fgf16* was involved in MHB development, we examined the expression of *fgf8* in *fgf16* morphants at 24 hpf. The expression of *fgf8* was detected in the MHB of *fgf16* morphants (n = 27/27) (Fig. 5A, B), which indicates that the MHB is normally formed in *fgf16* morphants.

We then investigated whether *fgf16* was involved in specification of the midbrain. Otx2 is an important player in the regulation of midbrain patterning [64], [65]. The expression of *otx2* was unaffected in the midbrains of *fgf16* morphants at 24 hpf (n = 13/13) (Fig. 5C, D). Furthermore, we investigated whether *fgf16* played a role in the specification of tectal and tegmental fates. The expression of *pax7a* and *nkx6.2* was also unaffected in the tectum and tegmentum in *fgf16* morphants at 24 hpf (n = 13/14 and n = 17/17), respectively (Fig. 5E–H). These results demonstrated that tectal and tegmental characteristics were not affected by *fgf16* MO, and also suggested that the morphological abnormalities observed in the midbrains of *fgf16* morphants may have been due to decreases in cell proliferation.

### *fgf16* was required for GABAergic neuron and oligodendrocyte development, but not for that of glutamatergic neuron

**Figure 6:**
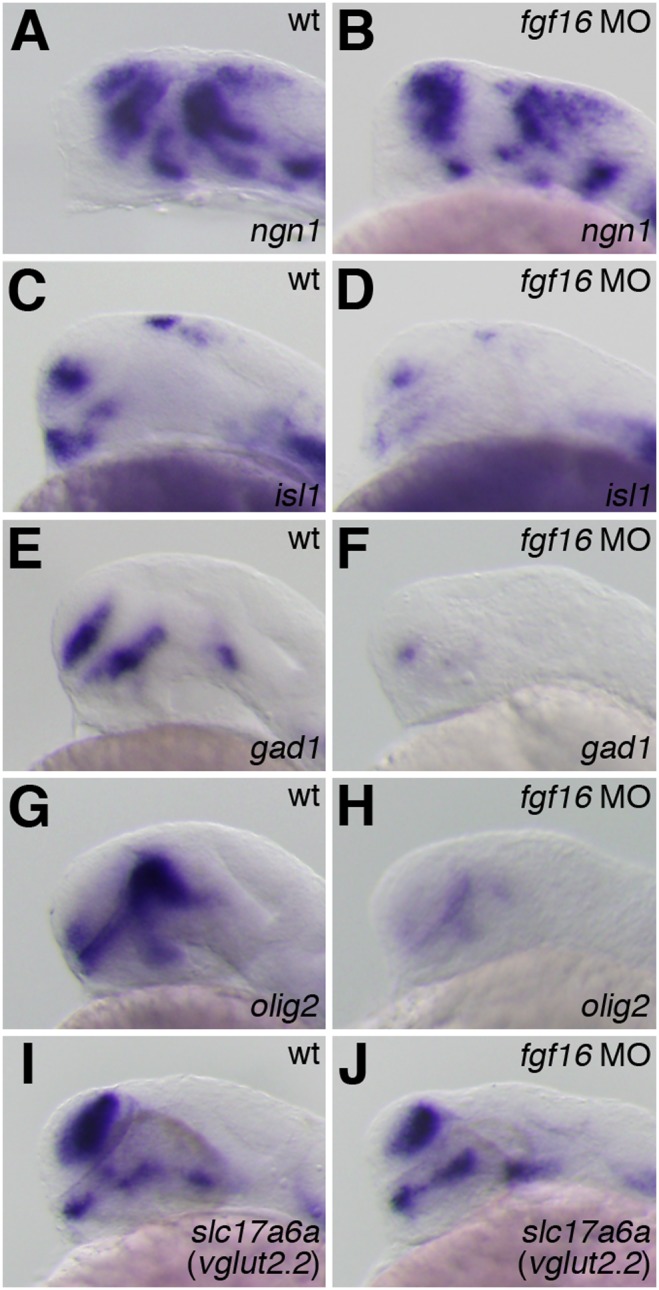
Effects of *fgf16* on the development of glutamatergic neurons, GABAergic interneurons, and oligodendrocyte progenitor cells. (A–D) The expression of *ngn1* (A, B) and *isl1* (C, D) in wild-type embryos (A, C) and *fgf16* morphants (B, D) at 24 hpf. Lateral views with anterior to the left and dorsal to the top. (E–J) The expression of *gad1* (E, F), *olig2* (G, H), and *slc17a6a* (I, J) in wild-type embryos (E, G, I) and *fgf16* morphants (F, H, J) at 28 hpf. Lateral views with anterior to the left and dorsal to the top.

In addition to patterning in the brain, Fgfs are involved in the development of neuronal subpopulations [8], [66], [67]. To determine whether an injection of *fgf16* MO affected neuronal differentiation in the forebrain, the expression of the basic helix-loop helix (bHLH) proneural gene, *ngn1*, was analyzed in *fgf16* morphants at 24 hpf. The expression of *ngn1* was unaffected in the dorsal telencephalon of *fgf16* morphants, whereas it was reduced in the diencephalon (n = 11/11) (Fig. 6A, B). We then examined whether the injection of *fgf16* MO affected the expression of *isl1*, a neuronal marker gene, in the forebrain. In the forebrain, *isl1* is expressed by ventral neurons in the telencephalon and diencephalon, and by neurons in the epiphysis at 24 hpf. The expression of *isl1* was reduced in the ventral telencephalon, anterior ventral thalamus, and epiphysis of *fgf16* morphants (n = 15/20) (Fig. 6C, D). These results indicated that neuronal differentiation in the ventral region in both the telencephalon and diencephalon was suppressed in *fgf16* morphants.

**Figure 7:**
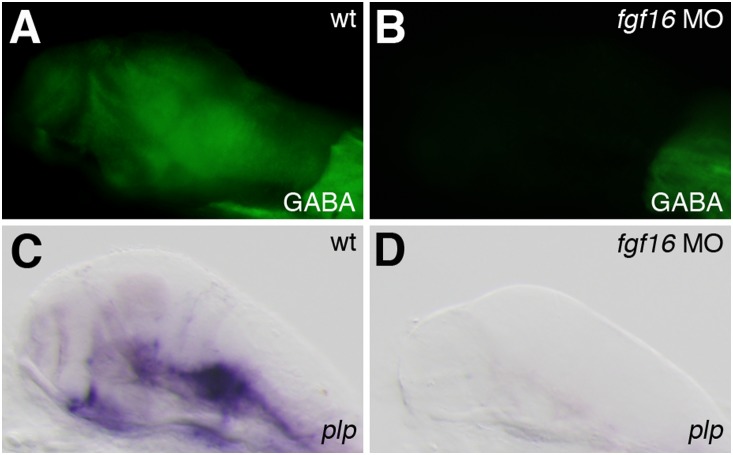
Effects of *fgf16* on the differentiation of GABAergic interneurons and oligodendrocytes. (A, B) Dorsal views of wild-type embryos (A) and *fgf16* morphants (B), labeled to show GABA immunoreactivity at 3 dpf. (C, D) The expression of *plp* in wild-type embryos (C) and *fgf16* morphants (D) at 4.5 dpf. Lateral views with anterior to the left and dorsal to the top.

GABAergic interneurons were previously shown to be generated in the subpallial telencephalon and ventral thalamus of the forebrain [68]–[71]. *gad1* encoding glutamic acid decarboxylase was found to be expressed specifically in GABAergic interneurons [42]. To examine whether the knockdown of *fgf16* had any effects on GABAergic interneuron differentiation in the forebrain, *gad1* expression was analyzed in *fgf16* morphants at 28 hpf. *gad1* was expressed in the subpallial telencephalon and nucleus of the tract of the postoptic commissure (nTPOC) in the forebrain [42]. In *fgf16* morphants, the expression of *gad1* was severely reduced in both the ventral telencephalon and the nTPOC (n = 27/28) (Fig. 6E, F). We also investigated whether GABAergic neurons fully differentiated in *fgf16* morphants. GABA-immunoreactive cells were not detected in the forebrains of *fgf16* morphants at 3 dpf (n = 20/20) (Fig. 7A, B). Oligodendrocytes in the telencephalon also originated from the subpallial domain [70]. To investigate the involvement of *fgf16* in oligodendrocyte specification, we examined the expression of *olig2*, a marker of the oligodendrocyte precursor, in *fgf16* morphants at 28 hpf. In addition to the subpallial telencephalon, *olig2* was also shown to be expressed in the ventral thalamus and dorsal thalamus [44]. In *fgf16* morphants, the expression of *olig2* was significantly reduced in the subpallial telencephalon, ventral thalamus, and dorsal thalamus (n = 14/20) (Fig. 6G, H). Furthermore, we determined whether *fgf16* was involved in the formation of myelinating oligodendrocytes. *PLP (proteolipid protein)/DM20* is a marker of oligodendrocyte differentiation and is expressed in newly formed oligodendrocyte progenitor cells, well before myelination [72]–[74]. The expression of *plp* was not detected in the forebrains of *fgf16* morphants at 4.5 dpf (n = 12/12) (Fig. 7C, D). In addition, the expression of *plp* in the hindbrain disappeared in *fgf16* morphants at 4.5 dpf (n = 10/12) (Fig. 7C, D). The immunoreactivity of CC1/APC, which is normally detected in mature oligodendrocyte cell bodies, was also lost in the hindbrains of *fgf16* morphants at 4.5 dpf (n = 11/11) (Fig. S2A, B). These results demonstrated that the specification and differentiation of GABAergic interneurons and oligodendrocytes in the forebrain was suppressed in *fgf16* morphants. We investigated whether the knockdown of *fgf16* had any effects on the differentiation of glutamatergic neurons generated in the pallial telencephalon [75]. The expression of *slc17a6a/vesicular glutamate transporter* (*vglut*) *2.2*, the postmitotic marker of glutamatergic neurons, was analyzed in *fgf16* morphants at 28 hpf. In *fgf16* morphants, the expression of *slc17a6a* was unaffected in both the pallial telencephalon and diencephalon (n = 14/14) (Fig. 6I, J). This result demonstrated that glutamatergic neurons in both the pallial telencephalon and diencephalon were specified in *fgf16* morphants. Thus, *fgf16* was required for the specification and differentiation of GABAergic neurons and oligodendrocytes, but not for that of glutamatergic neuron in the forebrain.

### Hh signaling was required for *fgf16* expression in the brain

**Figure 8:**
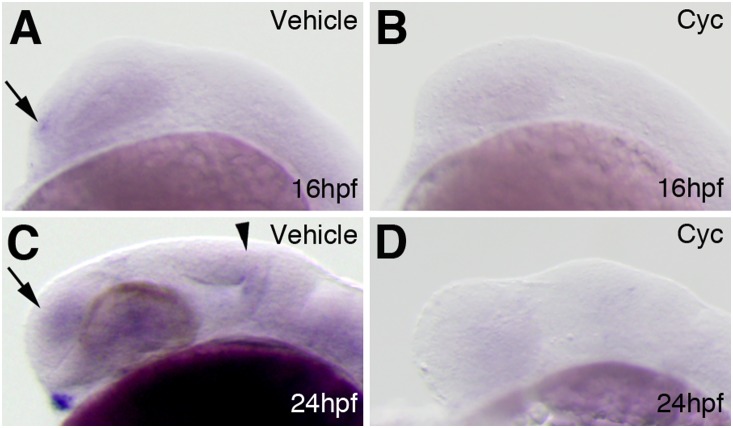
Interactions between *fgf16* and Hh signaling in the forebrain and midbrain. The expression of *fgf16* at 16 (A, B) and 24 (C, D) hpf in wild-type embryos treated with 0.95% ethanol (A, C) or cyclopamine (B, D). Arrows in panels A and C indicate *fgf16* expression in the telencephalon. The arrowhead in panel C indicates *fgf16* expression in the midbrain. Lateral views with anterior to the left and dorsal to the top.

Hh signaling in the ventral forebrain functions in dorsoventral (D/V) forebrain patterning and promotes the GABAergic neuronal/oligodendrocyte lineage restriction of forebrain stem cells [44], [76], [77]. The inhibition of *fgf16* led to abnormalities in the regionalization and generation of specific cell types such as GABAergic interneurons and oligodendrocytes in the forebrain. Hh signaling is critical for regulating the expression of *fgf3, fgf8*, and *fgf19* in the forebrain and that of *fgf19* and *fgf22* in the midbrain [8], [9]. Therefore, we examined whether the expression of *Fgf16* was responsive to Hh signaling. Since the alkaloid cyclopamine completely blocked Hh signaling at the level of Smoothened, which transduces Hh signals, in zebrafish [8], [78], we examined the expression of *Fgf16* in embryos treated with cyclopamine. In embryos treated with cyclopamine, *fgf16* expression was lost in the forebrain at 16 and 25 hpf (n = 16/16 and n = 10/10, respectively) (Fig. 8A–D). Furthermore, *fgf16* expression in the midbrain was lost in embryos treated with cyclopamine (n = 10/10) (Fig. 8C, D). All control embryos showed normal expression patterns for these genes (Fig. 8A, C). These results indicated that the expression of *Fgf16* in the forebrain and midbrain was dependent on Hh signaling.

### *fgf16* expression in the forebrain was lost in the *fgf19* morphant, but not in *fgf3/8* double morphant embryos

**Figure 9:**
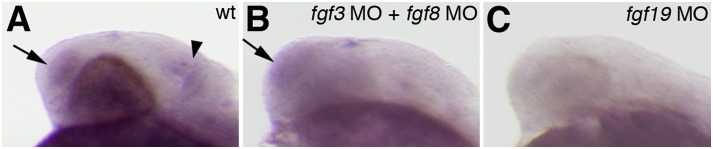
Interactions between *fgf3*, *fgf8*, *fgf19* and *fgf16*. The expression of *fgf16* at 24 hpf in wild-type embryos (A) and embryos injected with *fgf3* MO and *fgf8* MO (B), and *fgf19* MO (C). Arrows in panels A and B indicate *fgf16* expression in the telencephalon. The arrowhead in panel A indicates *fgf16* expression in the midbrain. Lateral views with anterior to the left and dorsal to the top.

The inhibition of *fgf16* led to abnormalities in the regionalization and generation of specific cell types such as GABAergic interneurons and oligodendrocytes in the forebrain. *fgf3* and *fgf8* are also involved in the regional patterning and generation of GABAergic interneurons and oligodendrocytes in the forebrain. The inhibition of both *fgf3* and *fgf8* was shown to result in defects in the expression of genes associated with early patterning functions and the specification of GABAergic interneurons and oligodendrocytes in the forebrain [7], [8], [18]. In the forebrain, the expression of *fgf16* was detected later than that of *fgf3* or *fgf8*. Therefore, to examine whether the expression of *fgf16* was affected by the inhibition of both *fgf3* and *fgf8* during forebrain development, we examined its expression in *fgf3/8* double morphant embryos at 24 hpf. The expression of *fgf16* was unaffected in the forebrains of *fgf3/8* double morphant embryos (n = 22/24) (Fig. 9A, B). In contrast to the forebrain, an injection of both *Fgf3* MO and *Fgf8* MO led to a reduction in the expression of *fgf16* in the midbrain at 24 hpf (n = 22/24) (Fig. 9A, B). This result indicated that the combinatorial function of *fgf3* and *fgf8* was involved in regulating *fgf16* expression in the midbrain, but not in the forebrain. In addition to *fgf3* and *fgf8*, *fgf19* is required for the regional patterning and specification of GABAergic interneurons and oligodendrocytes in the forebrain [8]. Furthermore, *fgf19* regulates the growth of the forebrain and midbrain [8]. The phenotype of *fgf16* morphants was essentially similar to that of *fgf19* morphants. Therefore, we also examined whether *fgf16* expression was affected in *fgf19* morphants. *fgf16* expression in both the forebrain and midbrain was reduced in *fgf19* morphants at 24 hpf (n = 12/14) (Fig. 9A, C). Thus, *fgf16* expression in the forebrain was regulated by the function of *fgf19*, but not by the combinatorial function of *Fgf3* and *Fgf8*. On the other hand, *fgf16* expression in the midbrain was dependent on *fgf3*, *fgf8*, and *fgf19*.

## Discussion

### Roles of *fgf16* in cell proliferation during brain development

Fgf signaling regulates the proliferation and differentiation of specific neuronal cell types in the forebrain and midbrain [8], [55], [56], [66]. *Fgf8* is required for MHB development, and the MHB is crucial for proliferation and patterning in the midbrain [60]–[63]. However, *fgf8* has not been implicated in growth of the forebrain [7]. On the other hand, *fgf16* knockdown significantly inhibited cell proliferation and led to a reduction in the size and morphological abnormalities in the forebrain and midbrain. *fgf16* morphants showed normal expression patterns of *fgf8* in the MHB and had normal MHB-specific characteristics. This result indicated that a decrease in cell proliferation in the midbrains of *fgf16* morphants was not due to a defect in the MHB. Thus, *fgf16* functions are required to promote cell proliferation in the forebrain and midbrain.

### Roles of *fgf16* in regional patterning during brain development

The expression of *fgf16* was first detected in the most anterior part of the ventral telencephalon at 14 hpf. *fgf16* morphants exhibited the expanded expression of markers for the pallial telencephalon, *emx1* and *tbr1,* and decreased expression of markers for the subpallial telencephalon, *dlx2,* at 24 hpf. These results suggested the loss of subpallial fate in the telencephalon of *fgf16* morphants. Reduced cell proliferation in the telencephalon was observed in *fgf16* morphants. Therefore, subpallial cells may be formed in smaller numbers due to reduced cell proliferation caused by the inhibition of *fgf16*. However, the expanded expression of *ngn1* and *slc17a6a* was not detected in the ventral telencephalon of *fgf16* morphants, which suggested that ventral cells in the telencephalon of *fgf16* morphants were not formed in smaller numbers. Furthermore, *fgf16* knockdown did not appear to transform cell fate specification from subpallial to pallial cells, and did not induce differentiation into dorsal neuronal cell types in the subpallial telencephalon. The ectopic expression of *otx2* was detected in the ventral telencephalon of *fgf16* morphants. Thus, Fgf16 is involved in patterning of the ventral forebrain, whereas the ventral telencephalon does not develop into the pallium following the inhibition of *fgf16*.

In the diencepharon, the expression of *dlx2* was decreased in the ventral thalamus by the inhibition of *fgf16* at 24 hpf, whereas that of *shh* was unaffected in the ventral region. Furthermore, the expression of *pax6a* was normally detected in the diencepharon of *fgf16* morphants at 24 hpf. These results demonstrated that the ventral thalamus was initially induced in *fgf16* morphants. Therefore, *fgf16* is necessary for maintaining of the characteristics of the ventral thalamus. In contrast, tectum- and tegmentum-specific characteristics were unaffected in the midbrains of the *fgf16* morphants. This result indicated that *fgf16* may be involved in regulating cell proliferation, but not dorsoventral patterning during midbrain development. In contrast, *fgf16* may be involved in both the establishment of the subpallial telencephalon and ventral thalamus as well as the regulation of cell growth during forebrain development.

### Roles of *fgf16* in specification of GABAergic interneurons and oligodendrocytes in the forebrain

Ngn1 is known to be sufficient for conferring neuronal identity on uncommitted precursors and plays an important role in neurogenesis [79]–[81]. Although Fgf signaling is involved in neuronal differentiation, the expression of *ngn1* was unaffected in the dorsal telencephalon of *fgf16* morphants. Furthermore, *slc17a6a* expression was also detected normally in the dorsal telencephalon of *fgf16* morphants. On the other hand, the expression of *isl1* was reduced in the ventral telencephalon, anterior ventral thalamus, and epiphysis, which suggested that *fgf16* may be involved in neuronal differentiation in the ventral region, but not the dorsal region in the forebrain. However, *slc17a6a* expression was detected normally in the ventral thalamus of *fgf16* morphants. These results indicated that *fgf16* was not required for the specification of glutamatergic neurons in the forebrain.

The expression of *dlx2* was reduced in the forebrains of *fgf16* morphants. Dlx2 was shown to be involved in the specification of GABAergic interneurons and oligodendrocytes in the telencephalon [82]. Dlx2 is known to induce the GABAergic marker, *GAD1*, when ectopically expressed in cortical explants [83]. *olig2*, expressed in oligodendrocyte precursors, is necessary and sufficient for the generation of oligodendrocytes throughout the neuraxis [44], [84], [85]. *fgf16* knockdown resulted in a severe reduction of the expression of *gad1* and *olig2* in the ventral telencephalon and diencephalon. GABA-immunoreactive cells were also lost in the forebrains of *fgf16* morphants, which indicated that GABAergic neurons did not fully differentiate in *fgf16* morphants. *plp* expression and CC1 immunoreactivity also disappeared in *fgf16* morphants, which suggested that the oligodendrocytes did not terminally differentiate into myelinating cells in *fgf16* morphants. These results demonstrated that *fgf16* was involved in the specification of GABAergic interneurons and oligodendrocytes in the ventral telencephalon and diencephalon. On the other hand, the knockdown of *fgf16* did not strongly stimulate apoptosis in the forebrain. This result suggested that the survival of GABAergic interneurons and oligodendrocytes was unaffected by *fgf16*. Accordingly, Fgf16 appears to be crucial for the differentiation of GABAergic interneurons and oligodendrocytes, but not for that of glutamatergic neurons in the forebrain.

### *fgf16* was regulated by Hh and *Fgf19* signaling in forebrain development

*Shh* plays a mitogenic role in the brain and the ectopic expression of Hh target genes causes human cancers such as Basal Cell Carcinoma or medulloblastoma, a granule cell tumor [58], [86], [87]. Cell proliferation in the forebrain and midbrain was decreased in the *fgf16* morphants as well as *Shh* mutant mice. Furthermore, the expression of *fgf16* in the forebrain and midbrain was markedly reduced by the inhibition of Hh signaling at 16 and 25 hpf. These results indicated that Fgf16 may function downstream of Hh activity in cell proliferation in the forebrain and midbrain. On the other hand, Fgf8 participates in the growth of the midbrain, whereas Fgf3 and Fgf8 are not required for growth of the forebrain [7], [18], [63]. Consistent with these findings, the inhibition of both *fgf3* and *fgf8* led to a reduction in the expression of *fgf16* in the midbrain, whereas it was unaffected in the forebrains of *fgf3/8* double morphant embryos. Thus, *fgf3* and *fgf8* expressed in the MHB may regulate cell proliferation in the midbrain by activating the expression of *fgf16* in the midbrain.

In addition to cell proliferation, Hh signaling is required for patterning in the telencephalon and the generation of GABAergic neuronal/oligodendrocyte progenitors from ventral forebrain stem cells via the activation of *olig2* [5], [44], [76], [77]. *fgf16* morphants as well as *smu/smo* mutants exhibited the suppressed specification of GABAergic interneurons and oligodendrocytes in the forebrain. Hh signaling specifies GABAergic interneurons and oligodendrocytes via *fgf3, fgf8*, and *fgf19* in the ventral forebrain, and this ensures the expression of pan-ventral transcription factors, such as *dlx2* and *olig2*, whereas Fgf19 has distinct functions independent from those of Fgf3 and Fgf8 [8]. The inhibition of *fgf19* led to a reduction in the expression of *fgf16* in the forebrain, whereas the expression of *fgf16* was unaffected in *fgf3/8* double morphant embryos. This result indicates that *fgf16* expression in the forebrain is regulated by Fgf19, but not by Fgf3/Fgf8. Thus, the effects of Hh activity on the differentiation of GABAergic interneurons and oligodendrocytes may be mediated through Fgf19-Fgf16 pathways in the forebrain.

In conclusion, the present results indicated that *fgf16* expressed in the developing brain plays crucial roles in brain development. *fgf16* is involved in cell proliferation in the forebrain and midbrain. *fgf16* is also involved in the development of the ventral region and specification and differentiation of GABAergic interneurons and oligodendrocytes in the forebrain. On the other hand, *fgf16* was not required for the specification of tectal and tegmental fates. Furthermore, the expression of *fgf16* was dependent on *Hh* and *fgf19*. The present results suggest that crosstalk between Fgf16 signaling and Fgf19 and Hh signaling may be crucial for cell proliferation, regionalization, and cell type specification during forebrain development.

## Supporting Information

Figure S1 Apoptosis in the brain of *fgf16* morphants. At 24 hpf, apoptotic cells in the brain of the wild-type (A) and *fgf16* MO1-injected (B) embryos were marked via TUNEL. Lateral views with anterior to the left and dorsal to the top.
(TIF)

Figure S2 Oligodendrocyte differentiation in the hindbrain of *fgf16* morphants. (A, B) Dorsal views of wild-type embryos (A) and *fgf16* morphants (B), labeled to show CC1/APC immunoreactivity at 4.5 dpf.
(TIF)
