# Supplementary figures and images for: Fgf16 Is Required for Specification of GABAergic Neurons and Oligodendrocytes in the Zebrafish Forebrain
Source: PLoS One. 2014 Oct 30;9(10):e110836. doi: 10.1371/journal.pone.0110836 (PMC4214708; doi:10.1371/journal.pone.0110836)

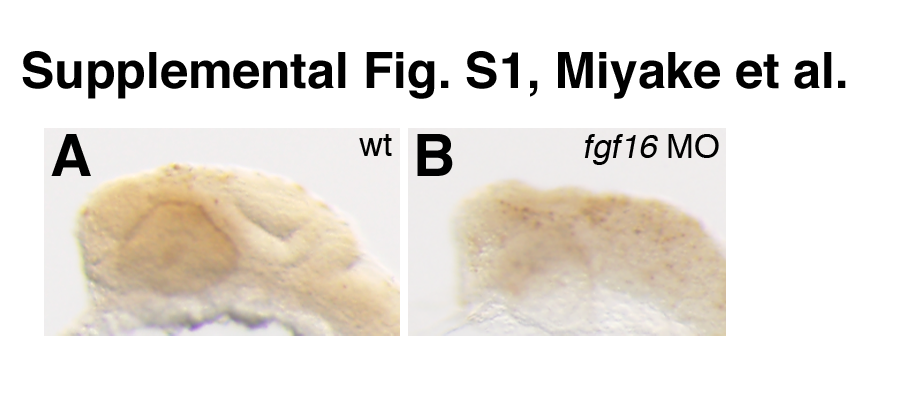

Supplement: Figure S1 — Apoptosis in the brain of fgf16 morphants. At 24 hpf, apoptotic cells in the brain of the wild-type (A) and fgf16 MO1-injected (B) embryos were marked via TUNEL. Lateral views with anterior to the left and dorsal to the top. (TIF) [file pone.0110836.s001.tif]

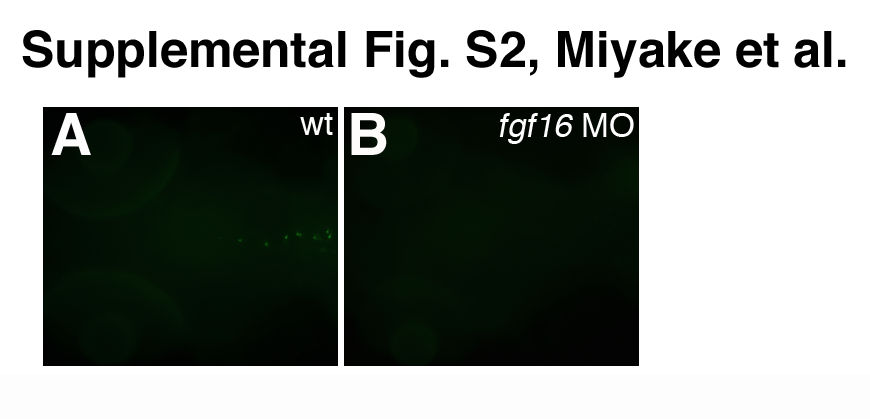

Supplement: Figure S2 — Oligodendrocyte differentiation in the hindbrain of fgf16 morphants. (A, B) Dorsal views of wild-type embryos (A) and fgf16 morphants (B), labeled to show CC1/APC immunoreactivity at 4.5 dpf. (TIF) [file pone.0110836.s002.tif]
